# Clinical, hormonal and metabolic parameters in women with PCOS with different combined oral contraceptives (containing chlormadinone acetate versus drospirenone)

**DOI:** 10.1007/s40618-019-01133-3

**Published:** 2019-10-25

**Authors:** A. Podfigurna, B. Meczekalski, F. Petraglia, S. Luisi

**Affiliations:** 1 Department of Gynecological Endocrinology, Poznan University of Medical Sciences, Poznan, Poland; 2 Department of Biomedical, Experimental and Clinical Sciences, Obstetrics and Gynecology, University of Florence, Florence, Italy; 3 Department of Molecular and Developmental Medicine, Obstetrics and Gynaecology, University of Siena, Siena, Italy

**Keywords:** Polycystic ovary syndrome, PCOS, Treatment, Oral contraceptives

## Abstract

**Introduction:**

Polycystic ovary syndrome (PCOS) is a common endocrine disorder affecting 5–10% of women of reproductive age. It is characterized by chronic anovulation leading to menstrual disorders, and increased infertility. The syndrome can also manifest as hirsutism and acne.

**Aim of the study:**

The aim of the study was to compare, over a duration of 6 months, the effects of drospirenone (DRSP) versus chlormadinone acetate (CMA) containing oral contraceptives (OCs) on clinical, hormonal, and metabolic parameters in 120 PCOS women.

**Materials and methods:**

120 women with the diagnosis of PCOS according to the Rotterdam 2003 criteria were recruited to the study. All patients were divided to two treatment groups of OCs, containing: 3 mg DRSP/30 mcg EE (ethinylestradiol) (60 patients) and 2 mg CMA/30 mcg EE (60 patients). Clinical parameters such as hirsutismus and acne were evaluated. Metabolic parameters such as serum insulin, glucose concentration, homeostatic model assessment of insulin resistance, body mass index, systolic and diastolic blood pressures were also measured. Among hormonal parameters, serum estradiol, luteinizing hormone, follicle-stimulating hormone, prolactin, testosterone, dehydroepiandrosterone sulfate, thyroid-stimulating hormone, and free thyroxine were measured.

**Results:**

The use of both DRSP- or CMA-containing OCs provided similar positive therapeutic effects with regard to clinical, metabolic, and hormonal parameters. Among clinical parameters, like hirsutismus, after 6 months of continuous OC treatment, a statistically significant improvement was observed in both groups: DRSP (*p* <  0.0001) and CMA OC treatment (*p* < 0.0001). In addition, significant improvement was showed according to acne lesions both after DRSP (*p* < 0.0001) and CMA treatments (*p* < 0.0001). Among glucose, insulin levels and HOMA-IR, there were statistically significant higher levels in both groups after DRSP (*p* < 0.0001, *p* < 0.0001, *p* < 0.05) and CMA OC treatment (*p* < 0.02, *p* < 0.0001, *p* < 0.0001). Hormonal parameters such as LH, FSH, prolactin, testosterone and DHEA-S were statistically significant lower in both groups after DRSP (*p* < 0.0001, *p* < 0.0001, *p* < 0.01, *p* < 0,002, and *p* < 0.0001) and CMA OC treatment (*p* < 0.0001, *p* < 0.0001, *p* < 0.04, *p* < 0.002, and *p* < 0.0001).

**Conclusions:**

Further research, however, is needed not only to define optimal duration, and to clarify the effects of treatment on long-term metabolic outcomes, but also to explore different treatment options and possible combined therapies.

## Introduction

Polycystic ovary syndrome (PCOS) is a common endocrine disorder affecting 5–10% of women of reproductive age. It is characterized by chronic anovulation leading to menstrual dysregulation (oligomenorrhea, amenorrhea), and increased infertility [1, 2].

The diagnosis of PCOS is based on the Rotterdam 2003 criteria that include the presence of two out of three of the following features: oligo/anovulation, clinical and/or biochemical hyperandrogenism, and polycystic ovaries by gynecological ultrasound [3]. Among hyperandrogenization symptoms, hirsutism is leading symptom. Other skin-related symptoms such as acne, seborrhea, androgenic alopecia are more rare in PCOS patients.

The endocrine profile of women with PCOS is characterized by high plasma concentrations of ovarian and adrenal androgens, gonadotropin abnormalities, a relative increase in estrogen levels (especially estrone) derived from the peripheral conversion of androgens, reduced serum levels of sex hormone-binding globulin (SHBG), and often high serum level insulin (INS) [4].

Hyperandrogenemia is a key feature of the syndrome, with excess androgens mainly of ovarian origin, although an adrenal source must always be considered as well. Most, but not all, women with PCOS have high plasma levels of androgens. Androstenedione (A) and testosterone (T) are markers of ovarian androgen secretion, and dehydroepiandrosterone sulfate (DHEAS) is the best marker of adrenal secretion. Hyperandrogenemia is not always apparent clinically. In certain patient groups, notably patients of Asian descent, hyperandrogenemia will often be present in the absence of superficial symptoms such as acne or hirsutism [5].

As the symptoms of PCOS accumulate and intensify, they can often lead to significant deterioration in quality of life, increase in stress, and negatively affecting psychological and sexual well-being [6–10].

The risk of comorbidities and long-term health consequences are higher among women with PCOS. These women generally have a higher chance of developing complications such as type II diabetes, obesity, hypertension, dyslipidemia and cardiovascular system diseases [11–14]. Zhang et al. discovered significantly higher serum irisin levels in women with the diagnosis of PCOS women compared with healthy women. Circulating concentrations of irisin were associated with hyperandrogenism, but not with oligoanovulation or PCO morphology [15]. According to Barry et al. meta-analysis, the risk of endometrial cancer in PCOS patients was significantly increased with odds (OR) 2.79 [16].

Insulin resistance is a common feature of PCOS and is more often seen in obese women, suggesting that PCOS and obesity have a synergistic effect on the magnitude of insulin dysregulation. Obesity leads to increased insulin secretion by beta-cells and a compensatory hyperinsulinemia. Insulin resistance with associated hyperinsulinemia has been causally linked to all features of PCOS, such as hyperandrogenism, reproductive disorders, acne, hirsutism and metabolic disturbances [17–20].

Women with PCOS have increased blood pressure, endothelial dysfunction, reduced arterial compliance, central obesity, dyslipidemia, low-grade chronic inflammation, and increased endothelin-1 and homocysteine [21].

The treatment of PCOS aims to reduce clinical features of the disorder and depends largely on the symptoms that are most troublesome to the patient. Response to therapy is slow, with biochemical reversal preceding clinical change by as much as 6–9 months. Treatment of PCOS requires a concerted effort on many fronts; non-pharmacological measures are universally recommended and include proper diet and exercise as weight reduction is critical if obese or when required to reestablish insulin sensitivity [22].

The most popular method of treating women with PCOS, with proven effectiveness, is the contraceptive pill, consisting of estrogens and progestogen [23]. The action of the contraceptive pill by inhibiting the hypothalamic-pituitary-ovarian axis causes regulation of the menstrual cycle in this group of women [24]. This type of treatment additionally has a positive effect on improving symptoms and hormonal parameters.

The most spectacular effect of the contraceptive pill seems to be the inhibition of androgen production by the ovaries [25, 26].

### Aim of the study

Compare clinical (hirsutism, acne), metabolic [glucose (GLU), insulin (INS), homeostatic model assessment of insulin resistance (HOMA-IR), systolic (SBP) and diastolic blood pressure (DBP), body mass index (BMI)] and hormonal [luteinizing hormone (LH), follicle-stimulating hormone (FSH), prolactin (PRL), testosterone (T), dehydroepiandrosterone sulfate (DHEA-S), thyroid-stimulating hormone (TSH), and free thyroxine (fT4)] parameters in women with PCOS following treatment with combined oral contraceptives—OCs containing either 3 mg Drospirenone (DRSP) or 2 mg Chlormadinone acetate (CMA) and 30 mcg Ethinyl estradiol (EE).

The aim of the study is based on comparison of DRSP versus CMA OCs treatment on metabolic and hormonal parameters in PCOS patients. The choice of described two hormonal preparations is based on our conscious choice. These two progestogens are classified to different progestagens groups and present different mechanism of action. Drospirenone (referred to fourth-generation progestins) presents mainly progestogenic, antimineralocorticoid and antiandrogenic effects. Chlormadinone acetate (is referred to first generation progestins) and presents progestagenic, antiandrogenic and weak glucocorticoid activity. From this point of view, two formulas of OC (CMA vs DRSP) theoretically can exert different effects on hormonal and metabolic parameters in PCOS patients.

## Experimental section

### Patients and methods

120 women with PCOS (age 26.92 ± 4.72 years) were recruited to the study. The diagnosis of PCOS was established based on the Rotterdam 2003 criteria [3]. All subjects were Caucasian, and were admitted to the Department of Gynecological Endocrinology, Poznan University of Medical Sciences, Poznan, Poland. Every subject was thoroughly interviewed by a physician, who collected a focused history covering gynecological, endocrine, psychiatric, and neurological health. Current illnesses, medications, allergies, recent weight changes, and menstrual details were also recorded. Patients receiving hormonal therapy (including OCs) or who had undergone hormonal therapy in the previous 6 months were excluded from the study. Patients reporting any chronic illness were also excluded. Weight and height of each subject was recorded, and BMI was calculated. Serum concentrations of E2, LH, FSH, PRL, T, DHEA-S, GLU, INS, TSH, and fT4 were measured by immunoenzymatic assay (ELISA). After overnight fasting, a blood sample was drawn from the cubital vein of each subject and collected in ethylenediaminetetraacetic acid-coated tubes between 7.30 a.m. and 9.00 a.m. Blood samples were drawn during the follicular phase (between the 8th and 12th day following the start of menstrual flow). Hirsutism was evaluated in each subject using the modified Ferriman and Gallwey scale (F–G scale) and acne lesions qualified subjectively on a scale between 0 and 3 points [27].

The modified F–G scale is a visual estimation method to determine hairiness in nine androgen-dependent body areas. This scale is limited by its subjective nature, but is currently the most widely recognized method of scoring hirsutism. The scale ranges from 0 to 36 points, with 8 points considered the minimal threshold for hirsutism.

Women were randomly assigned, using a random number scale, to one of two treatment groups of OCs, each, respectively, containing:

- 3 mg DRSP/30 mcg EE − 60 patients,
- 2 mg CMA/30 mcg EE − 60 patients.

Treatment was prescribed for a duration of 6 months (6 cycles counting 21 days hormone therapy followed by 7 days hormone free interval).

### Statistical analysis

STATISTICA 10 was used to analyze the data (StatSoft, United States of America). Student’s *t* test was used to compare means between treatment groups. Results were considered statistically significant at *p* < 0.05.

### Ethical considerations

All subjects provided informed consent to participate in the study before being included recruited. The study was conducted in accordance with the Declaration of Helsinki, and the study protocol was approved by the Ethics Committee of Poznan University of Medical Sciences, Poznan, Poland (project identification code: 516/16).

## Results

The parameters measured at baseline did not differ between the two study groups. Upon completing of the study, no significant differences were noted between the two study groups with respect to their selected end-points. Deviations from baseline of the measured parameters associated with treatment using DRSP and CMA OCs are indicated in bold in Tables 1, 2, 3, 4, 5, 6, 7, 8, 9.

**Table 1:** Clinical parameters (hirsutism and acne) in patients with PCOS before and after 6 months of DRSP treatment

Clinical parameter	Patients with PCOS before treatment	After 6 months of DRSP treatment	*p* value
Hirsutism (F–G scale)	17.86 ± 5.32	9.32 ± 4.98	< 0.0001
Acne (scale from 0 to 3)	2.32 ± 0.89	0.31 ± 0.78	< 0.0001

**Table 2:** Clinical parameters (hirsutism and acne) in patients with PCOS before and after 6 months of CMA treatment

Clinical parameter	Patients with PCOS before treatment	After 6 months of CMA treatment	*p* value
Hirsutism (F–G scale)	17.86 ± 5.32	9.55 ± 5.45	< 0.0001
Acne (scale from 0 to 3)	2.32 ± 0.89	0.45 ± 0.98	< 0.0001

**Table 3:** Comparison of clinical parameters (hirsutism and acne) in patients with PCOS after 6 months of DRSP treatment and after 6 months of CMA treatment

Clinical parameter	After 6 months of DRSP treatment	After 6 months of CMA treatment	*p* value
Hirsutism (F–G scale)	9.32 ± 4.98	9.55 ± 5.45	0.81
Acne (scale from 0 to 3)	0.31 ± 0.78	0.45 ± 0.98	0.39

**Table 4:** Metabolic parameters (glucose, insulin, HOMA-IR, systolic and diastolic blood pressure, BMI) in patients with PCOS before and after 6 months of DRSP OC treatment

Metabolic parameters	Patients with PCOS before treatment	After 6 months of DRSP OC treatment	*p* value
GLU (mg/dL)	86.46 ± 4.32	87.54 ± 4.98	0.21
INS (mU/mL)	17.89 ± 4.23	10.32 ± 5.01	< 0.0001
HOMA-IR	3.8 ± 0.19	2.2 ± 0.11	< 0.0001
SBP (mmHg)	128.42 ± 23.98	120.10 ± 21.76	< 0.05
DBP (mmHg)	82.71 ± 12.67	79.32 ± 13.23	0.15
BMI (kg/m^2^)	28.13 ± 5.79	26.34 ± 4.83	0.07

**Table 5:** Metabolic parameters (glucose, insulin, HOMA-IR, systolic and diastolic blood pressure, BMI) in patients with PCOS before and after 6 months of CMA OC treatment

Metabolic parameters	Patients with PCOS before treatment	After 6 months of CMA OC treatment	*p* value
GLU (mg/dl)	86.46 ± 4.32	88.45 ± 5.03	0.02
INS (mU/mL)	17.89 ± 4.23	11.53 ± 4.92	< 0.0001
HOMA-IR	3.8 ± 0.19	2.5 ± 0.12	< 0.0001
SBP (mmHg)	128.42 ± 23.98	119.20 ± 22.34	0.03
DBP (mmHg)	82.71 ± 12.67	78.39 ± 11.99	0.06
BMI (kg/m^2^)	28.13 ± 5.79	27.21 ± 4.92	0.35

**Table 6:** Comparison of metabolic parameters (glucose, insulin, HOMA-IR, systolic and diastolic blood pressure, BMI) in patients with PCOS after 6 months of DRSP OC treatment and after 6 months of CMA OC treatment

Metabolic parameters	After 6 months of DRSP OC treatment	After 6 months of CMA OC treatment	*p* value
GLU (mg/dL)	87.54 ± 4.98	88.45 ± 5.03	0.32
INS (mU/mL)	10.32 ± 5.01	11.53 ± 4.92	0.18
HOMA-IR	2.2 ± 0.11	2.5 ± 0.12	0.21
SBP (mmHg)	120.10 ± 21.76	119.20 ± 22.34	0.82
DBP (mmHg)	79.32 ± 13.23	78.39 ± 11.99	0.69
BMI (kg/m^2^)	26.34 ± 4.83	27.21 ± 4.92	0.33

**Table 7:** Hormonal parameters (E2, LH, FSH, PRL, T, DHEA-S, TSH, fT4) in patients with PCOS before and after 6 months of DRSP OC treatment

Hormonal parameters	Patients with PCOS before treatment	After 6 months of DRSP OC treatment	*p* value
Estradiol, E2 (pg/mL)	55.42 ± 21.65	56.67 ± 22.84	0.23
LH (mIU/mL)	13.66 ± 6.79	4.82 ± 3.28	< 0.0001
FSH (mIU/mL)	7.27 ± 1.87	4.98 ± 1.32	< 0.0001
PRL (ng/mL)	17.98 ± 5.23	16.56 ± 4.78	0.01
T (ng/mL)	0.75 ± 0.38	0.57 ± 0.23	0.002
DHEA-S (µmol/L)	11.01 ± 3.67	6.35 ± 2.78	< 0.0001
TSH (uIU/mL)	3.56 ± 1.29	3.54 ± 1.31	0.93
fT4 (ng/dL)	1.60 ± 0.43	1.59 ± 0.39	0.89

**Table 8:** Hormonal parameters (E2, LH, FSH, PRL, T, DHEA-S, TSH, fT4) in patients with PCOS before and after 6 months of CMA OC treatment

Hormonal parameters	Patients with PCOS before treatment	After 6 months of CMA OC treatment	*p* value
Estradiol, E2 (pg/mL)	55.42 ± 21.65	57.89 ± 23.12	0.19
LH (mIU/mL)	13.66 ± 6.79	4.78 ± 2.98	< 0.0001
FSH (mIU/mL)	7.27 ± 1.87	4.72 ± 1.92	< 0.0001
PRL (ng/mL)	17.98 ± 5.23	17.23 ± 3.97	0.04
T (ng/mL)	0.75 ± 0.38	0.56 ± 0.26	0.002
DHEA-S (µmol/L)	11.01 ± 3.67	6.98 ± 2.89	< 0.0001
TSH (uIU/mL)	3.56 ± 1.29	3.57 ± 1.33	0.97
fT4 (ng/dL)	1.60 ± 0.43	1.59 ± 0.41	0.90

**Table 9:** Comparison of hormonal parameters (E2, LH, FSH, PRL, T, DHEA-S, TSH, fT4) in patients with PCOS after 6 months of DRSP treatment and after 6 months of CMA OC treatment

Hormonal parameters	After 6 months of DRSP OC treatment	After 6 months of CMA OC treatment	*p* value
Estradiol, E2 (pg/mL)	56.67 ± 22.84	57.89 ± 23.12	0.24
LH (mIU/mL)	4.82 ± 3.28	4.78 ± 2.98	0.94
FSH (mIU/mL)	4.98 ± 1.32	4.72 ± 1.92	0.39
PRL (ng/mL)	16.56 ± 4.78	17.23 ± 3.97	0.41
T (ng/mL)	0.57 ± 0.23	0.56 ± 0.26	0.82
DHEA-S (µmol/L)	6.35 ± 2.78	6.98 ± 2.89	0.23
TSH (uIU/mL)	3.54 ± 1.31	3.57 ± 1.33	0.90
fT4 (ng/dL)	1.59 ± 0.39	1.59 ± 0.41	1.00

Clinical parameters of all patients with PCOS before treatment and in the two groups of patients after treatment are shown in Tables 1, 2, 3. Before treatment, all women suffered from hirsutism, with a mean score of 17.86 ± 5.32 points according to the F–G scale. After 6 months of continuous OC treatment, a statistically significant improvement was observed in both groups. Following treatment with DRSP, patients had a mean F–G score of 9.32 ± 4.98 (*p* < 0.0001), and following CMA OC treatment, a mean F–G score of 9.55 ± 5.45 (*p* < 0.0001) was recorded. No statistically significant difference in score was noticed between DRSP and CMA OCs groups after 6 months of treatment in women with PCOS (*p* = 0.81) (Table 3).

A secondary clinical parameter used for assessment was the severity of acne lesions. Before treatment, all women had been diagnosed with acne (mean score 2.32 ± 0.89 points). At end point, both DRSP and CMA treatment groups showed significant improvement: 0.31 ± 0.78 (*p* < 0.0001) and 0.45 ± 0.98 (*p* < 0.0001), respectively. A non-statistically significant difference was observed between the DRSP and CMA OCs treatment groups following 6 months of treatment in women with PCOS (*p* = 0.81) (Table 3).

The metabolic parameters of all patients before treatment and of the two patient groups after treatment are shown in Tables 4, 5, 6. Serum GLU and INS concentrations, HOMA-IR, SBP and DBP, and BMI were calculated. All these parameters were classified as metabolic parameters. The average fasting serum GLU concentration in patients with PCOS before treatment was 86.46 ± 4.32 mg/dL. After 6 months of DRSP OC treatment, serum GLU average level had increased (87.54 ± 4.98 mg/dL), but with no appreciable statistically significance (*p* = 0.21). After 6 months of CMA OC treatment, however, the average fasting serum GLU level had also increased (88.45 ± 5.03 mg/dL), a change that was calculated to be statistically significant (*p* = 0.02). When comparing the effects on serum GLU concentration between treatment groups, there was no statistical differences between treatment with DRSP and CMA OCs for 6 months in women with PCOS (*p* = 0.32).

The average serum INS concentration in women with PCOS before treatment was 17.89 ± 4.23 mU/mL. After 6 months of DRSP OC treatment, the average serum INS levels decreased significantly to 10.32 ± 5.01 mU/mL (*p* < 0.0001). Similarly, a statistically significant decline was observed in the group of patients treated with CMA OC: 11.53 ± 4.92 mU/mL (*p* < 0.0001). There was, however, no statistically significant difference in the serum INS levels between groups following treatment with DRSP and CMA OCs (*p* = 0.18).

Knowing how essential insulin resistance is as a metabolic parameter in PCOS, HOMA-IR was assessed in all patients. The average HOMA-IR score in women with PCOS recruited to this study was 3.8 ± 0.19. Among patients treated with DRSP OC, HOMA-IR after 6 months of treatment had decreased significantly to 2.2 ± 0.11 (*p* < 0.0001). The average HOMA-IR score in patients with PCOS after 6 months of CMA OC treatment had also decreased significantly to 2.5 ± 0.12 (*p* < 0.0001). When comparing both treatment groups, however, no statistically significant differences were observed in HOMA-IR score (*p* = 0.21).

The average SBP in patients before treatment was 128.42 ± 23.98 mmHg. After 6 months of DRSP OC treatment, a statistically significant decrease to 120.10 ± 21.76 mmHg (*p* < 0.05) was observed. Similarly, following 6 months of CMA OC treatment, a statistically significant decline in SBP to 119.20 ± 22.34 mmHg (*p* = 0.03) was observed. Once again, there was no statistically significant difference in SBP decline between groups following treatment with DRSP or CMA OC (*p* = 0.82).

The average DBP in patients before treatment was 82.71 ± 12.67 mmHg. Among patients treated with DRSP OC, the average DBP decreased to 79.32 ± 13.23 mmHg (*p* = 0.15) after 6 months of treatment; this change was not found to be statistically significant. The average DBP in patients following 6 months of CMA OC treatment also decreased to 78.39 ± 11.99 mmHg and was also not found to be significantly significant. When comparing both treatment groups, no statistically significant differences were observed between post-treatment DBP values (*p* = 0.21).

The final parameter that was assessed was BMI. The average BMI in women with PCOS before treatment was 28.13 ± 5.79 kg/m^2^. The average BMI calculated in the DRSP OC treatment group after 6 months of therapy was 26.34 ± 4.83 kg/m^2^ (*p* = 0.07), a change that was calculated to not be statistically significant. After 6 months of CMA OC treatment, the average BMI had also decreased, although even less significantly, to 27.21 ± 4.92 kg/m^2^ (*p* = 0.35). There was no statistically significant difference in BMI change between the treatment groups (*p* = 0.33).

Hormonal parameters of all patients before treatment and following treatment in one of the two treatment groups are shown in Tables 7, 8, 9. The average serum E2 level in patients with PCOS prior to treatment was 55.42 ± 21.65 pg/mL. After 6 months of DRSP OC treatment, serum E2 levels increased mildly, but not statistically significantly to 56.67 ± 22.84 pg/mL (*p* = 0.23). After 6 months of CMA OC therapy serum E2 level also increased (57.89 ± 23.12 pg/mL, *p* = 0.19), but as in the DRSP OC group, without statistical significance. The increases in serum E2 levels was not found to differ significantly between DRSP and CMA OC treatment groups (*p* = 0.24).

The average serum LH level in patients before treatment was 13.66 ± 6.79 mIU/mL. After 6 months of therapy, a statistically significant drop in serum LH was observed in both treatment groups. The DRSP OC group averaged 4.82 ± 3.28 mIU/mL serum LH, *p* < 0.0001, while the CMA OC group averaged 4.78 ± 2.98 mIU/mL (*p* < 0.0001) serum LH concentration. There was no statistically significant difference in post-treatment serum LH levels between the DRSP and CMA OC groups (*p* = 0.94).

The average serum FSH concentrations in subjects before treatment were 7.27 ± 1.87 mIU/mL. After 6 months of therapy, a decrease deemed statistically significant in serum FSH was observed in both treatment groups. The average serum FSH level had dropped to 4.98 ± 1.32 mIU/mL (*p* < 0.0001) in the DRSP OC group and 4.72 ± 1.92 mIU/mL (*p* < 0.0001) in the CMA OC group. When comparing inter-group serum FSH following treatment, no statistically significant differences were observed (*p* = 0.39).

The average baseline serum PRL level in patients included in this study was 17.98 ± 5.23 ng/mL. After 6 months of DRSP OC treatment, serum PRL levels fell to an average of 16.56 ± 4.78 ng/mL (*p* = 0.01). PRL levels in the CMA OC group fell to 17.23 ± 3.97 ng/mL (*p* = 0.04) following the same treatment time. There was no statistically significant difference in serum PRL following treatment in the DRSP or CMA OC groups (*p* = 0.41).

The average baseline serum T concentration in women participating in this study was 0.75 ± 0.38 ng/mL. Following 6 months duration of therapy, lower levels of T were observed on average, in both treatment groups. Serum T levels in the DRSP OC therapy group averaged 0.57 ± 0.23 ng/mL (*p* = 0.002). Similarly, the average serum T level in the CMA OC therapy group was 0.56 ± 0.26 ng/mL (*p* = 0.002). The average serum T concentration following treatment did not differ significantly between the DRSP and CMA OC groups (*p* = 0.82).

DHEA-S was also measured in the study participants. The average baseline serum DHEA-S concentration in women included in this study was 11.01 ± 3.67 µmol/L. After 6 months of treatment in both the DRSP OC therapy group and the CMA OC therapy group, statistically significant changes in average serum DHEA-S levels were observed. Subjects in the DRSP OC group had an average DHEA-S level of 6.35 ± 2.78 µmol/L (*p* < 0.0001) following therapy, whereas subjects in the CMA OC group averaged 6.98 ± 2.89 µmol/L (*p* < 0.0001). Differences in DHEA-S levels between groups following treatment, however, were not found to be statistical significant (*p* = 0.23).

Hormones of the thyroid gland were assessed in all patients participating in this study. The average serum TSH level at baseline for women entering this study was 3.56 ± 1.29 uIU/mL. Following 6 months of OC therapy, TSH levels were reevaluated, finding no statistically significant difference in their serum concentrations (3.54 ± 1.31 uIU/mL, *p* = 0.93 in the DRSP OC group and 3.57 ± 1.33 uIU/mL, *p* = 0.97 in the CMA OC group). Likewise, there was no appreciable difference in the effect of treatment on serum TSH concentration between groups (*p* = 0.90). The average serum fT4 level at baseline of women in this study was 1.60 ± 0.43 ng/dL. As with TSH levels, there was no significant change in fT4 levels following 6 months of OC therapy. Patients in the DRSP OC group had an average fT4 level of 1.59 ± 0.39 ng/dL (*p* = 0.89) following 6 months of treatment, whereas subjects in the CMA OC treatment group had an average fT4 level of 1.59 ± 0.41 ng/dL (*p* = 0.90). There was no significant difference in fT4 level change post-treatment between groups (*p* = 1.00).

## Discussion

When considering clinical parameters following treatment with both DRSP or CMA OCs in patients with PCOS, there was a statistically significant improvement both in the degree of hirsutism (as quantified by the F–G scale), and the level of acne reduction. No statistically significant difference was observed in the effect on clinical parameters between the two treatments.

When considering metabolic parameters, there was a statistically significant increase in serum GLU concentrations after 6 months of treatment with CMA OC. No such change was observed in the DRSP OC treatment group after 6 months. SBP and average serum INS levels were significantly lower after 6 months of treatment in both groups.

Average HOMA-IR score among subjects treated with DRSP or CMA OCs was significantly decreased when compared to baseline HOMA-IR. DBP and BMI, however, did not change significantly changed following hormonal therapy. Overall, there was no statistically significant difference in endpoint metabolic parameters between the DRSP and CMA OCs treatment groups.

Serum LH, FSH, PRL, T, and DHEA-S levels were all significantly decreased after 6 months of treatment in both the DRSP and CMA OC groups, while E2, TSH, and fT4 serum levels were not.

As was observed with the metabolic parameters, serum hormone concentrations at endpoint did not differ significantly between DRSP and CMA groups.

Estro-progestins are the primary treatment in PCOS. They act by reducing androgen synthesis, reducing serum androgen concentration and peripheral blocking activity in target tissues. OCs reduce the secretion of LH and FSH, thus decreasing ovarian androgen production and stimulate sex hormone-binding globulin (SHBG) production in the liver. As demonstrated in this study, estro-progestins reduced the amount of testosterone and DHEA-S levels. A decline in 5-α-reductase activity, causing a decrease in T to dihydrotestosterone conversion is also observed. Hyperandrogenism is best treated with antiandrogen-acting progestins. For example, gestagens such as DRSP or CMA have strong antiandrogenic effects [28].

Polycystic ovary syndrome, as a disorder rooted in multiple aspects of endocrine dysregulation, and it is often incomplete response to treatment, can be an extremely difficult problem to approach. With its proclivity for inducing negative body changes, and often lengthy and unsatisfactory therapeutic results, it can be a troublesome diagnosis for young patients. Young women with hyperandrogenism and unwanted body changed often independently seek symptom relief in cosmetic procedures. Unfortunately, such superficial treatments do not address the underlying hormonal imbalance, and often act to delay definitive treatment. Early diagnosis and treatment can suppress pathological processes associated with hyperandrogenism, and often prevent the development of a full-blown PCOS. Treating hirsutism in women is a delicate process and must always be appropriately balanced against the potential adverse effects of the medication used. This being said, many treatment options exist for both young girls, and adult women [29].

Our present data confirm the results published by Macut et al. [30] of a similar small group of women with PCOS. In their trial, patients were treated for 12 months with OCs containing EE and DRSP. The prolonged treatment in that study showed a decrease in serum androgens and increased cortisol in the presence of decreased hypothalamic-pituitary-adrenal axis sensitivity. These changes, however, did not alter glucocorticoid receptor (GR) expression and function.

Leelaphiwat et al. [31] did a comparison between the effects of EE 30 mcg/desogestrel 150 mcg plus spironolactone 25 mg/day, versus EE 35 mcg/cyproterone acetate 2 mg, demonstrating significantly decreased acne scores and free androgen index, while increasing SHBG levels. Cholesterol and high-density lipoprotein were significantly increased in patients treated with spironolactone, while androstenedione was significantly decreased in patients without spironolactone. Both regimens had similar efficacy in treating hyperandrogenism after three cycles of therapy, while having little effect on metabolic parameters. This study, however, utilized a relatively small sample size, which could well have impaired their detection ability. In a larger study group, such as the one presented in this paper (*n* = 120), significant and spectacular metabolic parameter changes were observed: both SBP and average serum INS levels were significantly decreased following 6 months of treatment.

Glintborg et al. [32] published a paper in which central obesity in PCOS was assessed. They also evaluated the association between PCOS and increased inflammatory markers, and estimated the risk for type 2 diabetes. The objective of the study was to evaluate whether treatment with metformin or metformin combined with OCs (desogestrel containing OCP) resulted in the development of more advantageous body composition when compared to treatment with OCs alone. 19 patients with PCOS were recruited to the study and treated for 12 months. Treatment with metformin and metformin with OCs were superior to OCs alone when considering outcomes of weight and regional fat mass distribution. OCs and metformin and oral contraceptive alone were superior to metformin alone at reduction of free T levels. Metformin treatment alone or in combination with OCs was associated with weight loss and improved body composition when compared with OCs alone. Free T levels decreased in subjects treated with metformin plus OCs or OCs alone. The study concluded that a combined treatment of metformin and OCs should be considered as an alternative to treatment with OCs alone to address weight gain in PCOS. In our study, BMI changes were observed in both groups following 6 months of DRSP or CMA treatment, however, these changes were not considered statistically significant.

Mes-Krowinkel et al. [33] evaluated the influence of OCs on anthromorphometric, endocrine, and metabolic parameters in women with PCOS. A total of 1297 patients were included. Anthromorphometric (blood pressure) and ultrasound parameters (follicle count, mean ovarian volume), endocrine [SHBG, T, free androgen index, antimüllerian hormone (AMH)], and lipid profiles were assessed. They observed that OC use contributes to a milder phenotypic presentation of PCOS with regards to hyperandrogenism; however, it does not alter parameters associated with increased health risks.

Kahraman et al. [34] compared the effects of OCs containing cyproterone acetate with those containing DRSP in the treatment of PCOS. 52 women were recruited to this study and treated for 12 months. The effects of both medication were similar on carbohydrate metabolism, lipid profile, and oxidative stress parameters. At the study endpoint, Cyproterone acetate containing OCs seems to be more effective in treating clinical hirsutism than DRSP OCs.

Di Carlo et al. [35] demonstrated the effects of 12-month treatment with an estradiol valerate/dienogest pill in 36 women suffering from PCOS with mild or moderate acne. The percentage of patients demonstrating regression of clinical symptoms was statistically significant. SHBG levels were significantly higher, while total serum T levels were lower in all patients at 6 and 12 months. The authors suggested that the estradiol valerate/dienogest pill could be beneficial in addressing acne and hyperandrogenism.

In October 2010, ESHRE/ASRM [36] PCOS workshop concluded: (1) all OCs appear to have equal efficacy in treating PCOS, (2) addition of an antiandrogen (spironolactone) to OCs has little treatment benefit, and (3) metformin does not improve live-birth rates and should only be used in patients with impaired glucose tolerance. The cohort which served to issue these statements counted 1.6 million women taking OCs, 46,780 of which were identified as having PCOS. Their data suggested that further attention was needed in the medical management of PCOS and in bridging the gap between guidelines and practice.

Bhattacharya et al. [37] compared the effect of OCs containing desogestrel, cyproterone acetate, and D in women with PCOS. Treatment outcomes were assessed at 6 and 12 months. The study was structured as a double-blind randomized controlled trial. 171 women previously diagnosed with PCOS were recruited. BMI, abdominal circumference, hirsutism score (modified F–G scale), acne, acanthosis nigricans, and blood pressure were noted. Blood serum levels of total T, SHBG, fasting GLU, and fasting INS were measured. Free androgen index, GLU-INS ratio, and homeostasis model assessment-insulin resistance (HOMA-IR) were calculated. There was no difference in the effect of treatment between 6 and 12 months. At the study endpoint, cyproterone acetate was shown to have the strongest antiandrogen effect.

Aydin et al. [38] evaluated the effects of OCs containing DRSP on body composition in lean women with PCOS. The authors recruited 28 subjects who were treated with EE 30 mcg/D 3 mg for a duration of 6 months. Body composition parameters were assessed by bioelectrical impedance analysis. Serum androgens and lipids, INS resistance, and GLU metabolism were also assessed. Lean women with PCOS have similar body composition compared to healthy women. OCs therapy for 6 months in the subjects, however, resulted in an increased total and trunk fat percentage, despite no appreciable change in clinical anthropometric measurements including weight, BMI, and Waist–Hip Ratio (WHR).

Another interesting study was conducted by Bhattacharya et al. [39] where the effects of 30 μg and 20 μg ethinyl estradiol (EE) were compared in 112 women with PCOS. Following 6 months of treatment, the free androgen index had decreased by 4.96 ± 6.01 in patients receiving 30 μg EE (*n* = 55) and by 4.81 ± 6.03 in those receiving 20 μg EE (*n* = 57; *p* = 0.89). At 12 months, the decrease from baseline was 5.23 ± 5.79 and 4.99 ± 5.86 (*p* = 0.82) respectfully. The authors concluded there was no difference in the change in androgen levels among subjects using an oral contraceptive pill containing 20 μg EE and 30 μg EE.

In the world-wide literature, Yildishan study [24] is the only study concerning also comparison of CMA and DRSP OCs use in PCOS patients. Our study is second in the world-wide literature which concerns this topic. Therefore, from this point of view, our study can be regarded as novel, although treatment is based on two commonly used combined oral contraceptives. Additionally, novelty is related to the facts that our study was done on Polish population of young women with PCOS. Yildishan study was performed on Turkish PCOS population. Our study evaluates short-term (a 6-month treatment) in comparison to 24-month treatment (Yildizhan [24]); as we know from the literature, the 6-month treatment is typical time after which we can assess efficacy of the OC therapy. Our study presents more extensive hormonal (thyroid function and adrenal function) evaluation after the treatment than Yildizhan study.

Yildizhan study revelaed that DRSP OC formula is more favorable than CMA OC formula favorable in the contex better effect on lipid profile, hsCRP levels, insulin resistance and hyperandrogenism. This is general conclusion from this study which evaluated mentioned parameters after 6, 12 and 24 months of treatment.

In our study, we did not find differences between two types of OC treatment impact on hyperandrogenization (hirsutism, acne), insulin resistance, blood pressure and hormonal (thyroid and adrenal) function.

However, in Yildizhan study, after 6 months of treatment more beneficial effect of DRSP was observed only in the aspect of total cholesterol and hsCRP and free androgen index (FAI).

Other parameter such as insulin resistance (HOMA-IR) did not change after 6 months of both treatments (similarly as in our study).

In 2018, another study interesting a study was published by Jaisamrarn [40]. They compared combined oral contraceptives containing CMA versus DRSP for the treatment of acne and dysmenorrhea in Thailand population (non-PCOS patients). They found that CMA OCs are more effective for the treatment of acne and dysmenorrhea in women with moderate acne and dysmenorrhea than DRSP OCs. In our study, we did not observe such finding concerning acne treatment with CMA and DRSP OCs.

Estrogen component (in our study ethinylestradiol) of oral contraceptives can potentially influence the thyroid function. It is referred to increase the serum concentrations of different binding proteins for instance thyroxine-binding globuline (TBG). It can cause the rise of total thyroxine. Wiegratz et al. [41] studied influence of four oral contraceptives on thyroid and adrenal parameters. They found that three dienogest-containing and levonorgestrel-containing low doses OC may increase total T3 and T4 (and cortisol), while the free proportion of hormones is only slightly changed. The number of studies evaluating influence of CMA and DRSP containing OC on thyroid function is very limited, therefore, we decided to assess this hormonal aspect.

## Conclusions

In this study, we present the outcome of a relatively large group of patients treated for PCOS using one of two OCs. Many parameters were assessed, including clinical, metabolic, and hormonal. Among clinical parameters, hirsutism and acne were monitored. Metabolic parameters such as serum glucose, insulin levels, HOMA-IR, SBP and DBP and BMI were measured and analyzed. Hormonal parameters included E2, LH, FSH, P, T, DHEA-S, TSH, and fT4. Two OC medications were evaluated, both providing satisfactory relief of symptoms and statistically significant outcomes in almost all evaluated parameters. Following treatment, the differences in outcome between DRSP and CMA OCs at 6 months were not considered statistically significant. Based on these findings, we conclude that the use of either DRSP or CMA containing OC treatment provides equivalent therapeutical benefit.

Further research is still needed to define the optimal duration of treatment and to better establish the effects of treatment on long-term metabolic outcomes. Future research should also focus on incorporating varied treatments as well as combined therapies.
